# Surface cysteine to serine substitutions in IL-18 reduce aggregation and enhance activity

**DOI:** 10.7717/peerj.13626

**Published:** 2022-07-05

**Authors:** Jirakrit Saetang, Niran Roongsawang, Surasak Sangkhathat, Supayang Piyawan Voravuthikunchai, Natnaree Sangkaew, Napat Prompat, Teerapol Srichana, Varomyalin Tipmanee

**Affiliations:** 1 International Center of Excellence in Seafood Science and Innovation, Faculty of Agro-Industry, Prince of Songkla University, Hat Yai, Songkhla, Thailand; 2 Department of Surgery, Faculty of Medicine, Prince of Songkla University, Hat Yai, Songkhla, Thailand; 3 EZ-Mol-Design Laboratory, Faculty of Medicine, Prince of Songkla University, Hat Yai, Songkhla, Thailand; 4 Microbial Cell Factory Research Team, Biorefinery and Bioproduct Technology Research Group, National Center for Genetic Engineering and Biotechnology, National Science and Technology Development Agency, Khlong Luang, Pathum Thani, Thailand; 5 Department of Biomedical Sciences and Biomedical Engineering, Prince of Songkla University, Hat Yai, Songkhla, Thailand; 6 Translational Medicine Research Center, Faculty of Medicine, Prince of Songkla University, Hat Yai, Songkhla, Thailand; 7 Center of Antimicrobial Biomaterial Innovation-Southeast Asia and Natural Product Research Center of Excellence, Faculty of Science, Prince of Songkla University, Hat Yai, Songkhla, Thailand; 8 Drug Delivery System Excellence Center and Department of Pharmaceutical Technology, Faculty of Pharmaceutical Sciences, Prince of Songkla University, Hat Yai, Songkhla, Thailand

**Keywords:** Interleukin-18, Surface cysteine, Aggregation, Interferon-γ, Molecular dynamic simulation

## Abstract

**Background:**

Interleukin-18 (IL-18) is prone to form multimers resulting in inactive aggregates, making this cytokine unstable for clinical use. Therefore, mutations have been introduced into recombinant IL-18 to overcome this issue.

**Methods:**

To prevent the formation of disulfide bonds between the IL-18 molecules, multiple mutations targeting surface cysteines (C38, C68, C76, and C127) were introduced into our previously modified human IL-18 double mutant E6K+T63A (IL-18 DM) by direct gene synthesis. The open reading frames of IL-18 wild-type (WT), IL-18 DM, and IL-18 multiple mutant E6K+T63A+C38S+C68S+C76S+C127S (IL-18 DM1234) were inserted in the pET28a expression vector and transformed into *Escherichia coli* Rosetta2 (DE3) pLysS cells for protein production. The inclusion bodies of WT and mutated IL-18 were extracted by sonication and refolded by stepwise dialysis using 8 M urea as the starting concentration. The refolded IL-18 proteins were tested for aggregation using the ProteoStat protein aggregation assay. Their activity was also investigated by treating NK-92MI cells with each IL-18 at concentrations of 75, 150, and 300 ng/ml with 0.5 ng/ml of human IL-12 and interferon-gamma (IFN-γ) levels in the supernatant were evaluated using ELISA. The structure of modified IL-18 was visualized using molecular dynamics (MD) simulations.

**Results:**

IL-18 DM1234 exhibited the lowest aggregation signal, approximately 1.79- and 1.63-fold less than that of the WT and IL-18 DM proteins. Additionally, the IFN-γ inducing activity of IL-18 DM1234 was about 10 and 2.8 times higher than that of the WT and IL-18 DM, respectively. MD simulations revealed that binding site I of IL-18 DM1234 was altered mainly due to surface cysteine replacement with serine (C-to-S substitution). This is the first report showing that C-to-S substitutions in IL-18 improved its activity and stability, suggesting the use of this modified IL-18 for medical purposes in the future.

## Introduction

Interleukin-18 (IL-18) is a cytokine that plays a key role in many aspects of the human immune system. This cytokine was previously named the interferon-γ-inducing factor, reflecting its function in the human physiology (Okamura et al., 1995). Currently, IL-18 is recognized as a cytokine that promotes the type 1 helper T cell response, which is essential for anti-tumor immunity (Fabbi, Carbotti & Ferrini, 2015; Nakamura et al., 2018). Moreover, IL-18 polarizes human natural killer (NK) cells to develop a distinct helper differentiation phenotype (CD83^+^CCR7^+^CD25^+^) and stimulates cytotoxic lymphocytes to enhance interferon-gamma (IFN-γ) secretion, granule-mediated cytotoxicity, and Fas ligand expression (Mailliard et al., 2005; Li et al., 2021). Therefore, clinical studies using IL-18 in humans have been conducted (Robertson et al., 2006; Robertson et al., 2008; Briukhovetska et al., 2021). However, although no serious adverse effects were found in cancer patients treated with IL-18, the efficacy was still limited due to increased expression of its high-affinity antagonist IL-18-binding protein (IL-18BP) in several cancers (Fabbi, Carbotti & Ferrini, 2015; Nakamura et al., 2018). In addition, precipitation or aggregation, commonly observed in recombinant cytokine therapeutics, is another problem that may cause loss of activity and increase the risk of immunogenicity (Lipiäinen et al., 2015).

To solve these problems, several molecular engineering studies on this protein have been attempted through site-directed mutagenesis and protein engineering. The substitution of glutamic acid to alanine at position 6 (E6A) and lysine to alanine at position 53 (K53A) in IL-18 enhanced its activity and reduced its affinity toward IL-18BP (Kim et al., 2001). In a similar report, mutation of E6 to lysine (E6K) increased the activity of IL-18, resulting in IFN-γ induction by approximately 3-8 times higher than wild-type (WT) IL-18 (Kim et al., 2002). Another study highlighted that replacing the intramolecular core threonine at position 63 with alanine enhanced IL-18 activity by approximately three times compared to that of WT IL-18 (Swencki-Underwood et al., 2006). Recently, a decoy-resistant IL-18 was developed for application in cancer immunotherapy. This modified IL-18 was reported to be less sensitive to IL-18BP than WT IL-18 and promoted the polarization and stimulation of effector cytotoxic T cells and NK cells, respectively (Zhou et al., 2020). While most studies have focused on improving IL-18 activity, some have attempted to develop low-aggregating IL-18. For example, cysteine residues at the surface of IL-18 were reported to be associated with protein aggregation and instability, and their replacement with serine at positions 38, 68, 76, and 127 made IL-18 tolerant to oxidative stress and decreased its aggregation (Yamamoto et al., 2004).

In a previous study, we developed a modified human IL-18 double mutant E6K+T63A with approximately 16 times higher activity than that of the WT protein (Saetang et al., 2016). It was found to be a potent inducer of IFN-γ in NK-92MI cells. Moreover, it showed anti-tumor activity that promoted type 1 helper T cell and cytotoxic T lymphocyte response in a CT26-WT colon cancer animal model (Saetang et al., 2020). However, according to stability testing, IL-18 tends to form multimers, leading to inactive IL-18 aggregates (Li et al., 2003; Yamamoto et al., 2004). Aggregation of modified IL-18 at higher concentrations was also observed in our study.

Therefore, to reduce aggregation and improve the stability of the modified recombinant IL-18, we attempted to reduce the intermolecular interactions between IL-18 molecules by replacing surface cysteines with serine (C-to-S substitutions). This method successfully enhanced the solubility of WT human IL-18 in a previous study (Yamamoto et al., 2004). Likewise, the C-to-S substitutions in this study improved the solubility and activity of modified IL-18, revealed during protein production and subsequent activity testing. Molecular dynamics (MD) simulations were also performed for structural analysis of IL-18.

## Materials and Methods

### Plasmid construction and protein expression

All plasmids were synthesized and obtained from GenScript (New Jersey, USA). The open reading frame (ORF) of the WT IL-18, codon-optimized for *E. coli* Rosetta2 (DE3) pLysS expression system, was inserted between *Nde* l and *Eco* RI restriction sites of the pET28a expression vector. The IL-18 double mutant E6K+T63A (IL-18 DM) and IL-18 with multiple mutations E6K+T63A+C38S+C68S+C76S+C127S (IL-18 DM1234) were also obtained from the same source, cloned using similar restriction sites in the pET28a vector. All recombinant plasmids (pET28a-IL-18WT, pET28a-IL-18DM, and pET28a-IL-18DM1234) were transformed into *E. coli* Rosetta2 (DE3) pLysS cells and selected on Luria Bertani (LB) agar (Sigma-Aldrich, St. Louis, MO, USA) containing 34 mg/ml chloramphenicol (Invitrogen, Waltham, MA, USA) and 50 mg/ml kanamycin (Invitrogen, Waltham, MA, USA). Further, the selected clones were used for IL-18 production. Briefly, the recombinant clones bearing WT and mutated IL-18 were cultivated in LB broth supplemented with 34 mg/ml chloramphenicol and 50 mg/ml kanamycin and incubated in a shaker incubator (Wiggens, Straubenhardt, Germany) at 37 °C, 200 rpm until OD600 reached 0.6. Then, 1 M IPTG (isopropyl β-D-1-thiogalactopyranoside; Sigma-Aldrich, St. Louis, MO, USA) was added to the culture at a final concentration of 0.1 M and incubated at 25 °C, 200 rpm in a shaker incubator for 5 h. Post-production, cells were harvested by centrifugation at 10,000 rpm for 10 min at 4 °C and resuspended in 10 ml PBS for sonication (on ice) with 60% amplitude, pulsed at 10 s on/15 s off for 5 min with breaks between cycles to prevent warming of the mixture. The cell lysate was then centrifuged for 10 min at 10,000 rpm at 4 °C. The supernatant and cell pellet were separated and analyzed on 12% SDS-PAGE.

### Step-wise refolding

The sonicated cell pellet was washed with 25 ml PBS thrice and centrifuged at 10,000 rpm for 10 min. The pellet was then collected, weighed, and resuspended in solubilization buffer (base buffer (50 mM Tris-HCl, 150 mM sodium chloride (NaCl), 100 mM glycine, 20% glycerol, pH 8.0) containing 8 M urea) at a final concentration of 2–8 mg/ml. The suspension was mixed in an orbital shaker for 30 min, and after centrifugation at 15,000 rpm for 15 min, the supernatant was filtered through a 0.45 µm syringe filter. The supernatant was dialyzed using slide-A-Lyzer G2 dialysis cassettes (Thermo Scientific, Waltham, MA, USA) in 1 L dialysis buffer (base buffer containing 6 M urea) for 4 h at 4 °C. During this step, 500 mL of base buffer was added to the dialysis buffer every 6 or 12 h 4 times. Finally, the dialysis cassette with the solubilized IL-18 inclusion bodies was placed in 2 ml base buffer for 6 h. The refolded IL-18 proteins (WT and mutants) were harvested and stored at 4 °C until further use.

### Western blotting

The proteins obtained after running on a 12% SDS-PAGE gel transferred onto a polyvinylidene difluoride (PVDF) membrane (Bio-Rad, Hercules, CA, USA) by electroblotting at 35 volts for 16 h in transfer buffer (25 mM Tris, 192 mM glycine, 20% methanol, pH 8.3). Non-specific proteins were blocked by incubating the PVDF membrane in Tris-buffered saline plus 0.1% Tween 20 (TBST) containing 3% (w/v) bovine serum albumin (BSA) (Sigma-Aldrich, St. Louis, MO, USA) for 1 h at 25 °C, followed by washing with TBST thrice. The membrane was then stained with a specific mouse anti-human IL-18 antibody (R&D Systems, Minneapolis, MN, USA; catalog number D043-3) at 1:3,000 dilution for 1 h at 25 °C and washed three times for 5 min each in TBST with gentle agitation. In the next step, horseradish peroxidase-conjugated goat anti-mouse IgG (R&D Systems, Minneapolis, MN, USA; catalog number HAF007) was added at a dilution of 1:10,000 in TBST containing 3% (w/v) BSA, and the membrane was incubated for 1 h at 25 °C. After washing three times in TBST, IL-18 was detected using the Luminata Forte Western HRP substrate (Millipore, Burlington, MA, USA) with the UVITEC Cambridge Gel Documentation System (UVITEC, Cambridge, UK).

### Liquid Chromatography with tandem mass spectrometry (LC-MS/MS)

LC-MS/MS analysis was performed using the commercial service of the Proteomics International Company, Australia. Protein samples were trypsin-digested, and peptides were extracted according to standard techniques (Bringans et al., 2008). The peptides were analyzed by electrospray ionization mass spectrometry using a Shimadzu Prominence nano HPLC system (Shimadzu, Kyoto, Japan) coupled to a 5600 TripleTOF mass spectrometer (Sciex, Redwood City, CA, USA). Tryptic peptides were loaded onto an Agilent Zorbax 300SB-C18, 3.5 µm (Agilent Technologies, Santa Clara, CA, USA) and separated with a linear gradient of water/acetonitrile/0.1% formic acid (v/v). Spectra were analyzed to identify the proteins of interest using Mascot sequence matching software (Matrix Science, UK) with the UniProt database.

### Protein aggregation assay

The WT and mutated IL-18 proteins were subjected to buffer exchange using a Vivaspin ultrafiltration unit (Sartorius, Göttingen, Germany) against PBS. To generate IL-18 aggregates, 40 µg/ml of each protein was incubated at 37 °C, 220 rpm for 16–18 h in a shaker incubator. The protein aggregation was monitored by ProteoStat Protein Aggregation Assay (Enzo Life Sciences, Farmingdale, NY, USA) according to the manufacturer’s protocol. Briefly, the proteins were loaded in triplicate in 96 well fluorescent microplates, followed by the addition of ProteoStat detection dye. The reaction mixture was then incubated for 15 min in the dark at room temperature. The fluorescence signal was determined by excitation at 485 nm and emission at 620 nm using a Varioskan™ LUX multimode microplate reader (Thermo Scientific, USA).

### IFN-γ induction assay

For the IFN-γ induction assay, the NK-92MI cell line was selected because it is a highly cytotoxic IL-2 independent NK cell line that expresses various types of cytokines (Yan et al., 1998; Tam et al., 1999). The cell line was purchased from the American Type Culture Collection (ATCC, Manassas, VA, USA) and maintained in complete α-minimum essential medium (MEM) supplemented with 12.5% fetal bovine serum (FBS) and 12.5% horse serum at 37 °C in a CO_2_ incubator. The cell line was used to evaluate the activity of WT and mutated IL-18, and the experiment was performed as previously described (Saetang et al., 2016). Briefly, the NK-92MI cells were suspended in 0.2 ml complete α-MEM at 0. 5 × 10^6^ cells/ml. Then, IFN- γ was induced using each recombinant IL-18 protein at concentrations of 75, 150, and 300 ng/ml, respectively, with 0.5 ng/ml of human IL-12. After 16–18 h of incubation at 37 °C in a CO_2_incubator, the supernatants were collected, and IFN-γ levels were estimated using the Human IFN-γ Quantikine ELISA Kit (R&D system, Minneapolis, MN, USA).

### MD simulations

The crystal structure of human IL-18 (accession code 3WO2) was taken from the RCSB Protein Data Bank (PDB) (Tsutsumi et al., 2014). The co-crystallized solvents were removed from the structure. The cysteine-serine replacement was performed using the FoldX package (Schymkowitz et al., 2005). All ionizable amino acids were set to their default state at pH 7.0. Neutral histidine was set as a single ϵ-protonated histidine (HIE in AMBER name). Double protonated histidine or disulfide linkages were not observed. The IL-18 protein was then solvated using the TIP3P water rectangular box at a distance of 14 Å from the protein surface. The system was neutralized using sodium ions, and NaCl was added to create a 0.15 mol L^−1^ NaCl solution (Saetang et al., 2016) using the AMBER (Assisted Model Building with Energy Refinement) 20 force field (Case et al., 2021) via the Leap module in the AMBER20 package. In total, the system consisted of monomeric IL-18, 4 sodium ions, 33 NaCl pairs, and 11437 TIP3P waters.

WT and mutated IL-18 structures were energy-minimized using steeping descent and conjugate gradient methods for 1,000 steps each under periodic boundary conditions. The minimized structures were subjected to canonical (NVT) simulation at 37 °C (310 K), where all protein positions were restrained by a harmonic potential. Temperature was controlled using a Langevin thermostat. All non-bonded and electrostatic interactions were calculated using a cutoff of 16 Å. Each NVT restrained simulation was executed for 200 picoseconds (ps) with a time step of 1 femtosecond (fs) and force constants of 200, 100, 50, 20, and 10 kcal/mol Å-2 in a total of 1 nanosecond (ns). To apply a pressure of 1.013 bar (1 atm), the system was changed to an isobaric-isothermal (NPT) simulation. Temperature and pressure were regulated using a weak-coupling algorithm (Berendsen et al., 1984). The NPT simulation lasted 120 ns, with a time step of 2 fs. The first 60 ns simulation was omitted, and the last 60 ns simulation was taken into the analyzed trajectory. All energy minimizations and molecular dynamics processes were performed using the Particle Mesh Ewald Molecular Dynamics (PMEMD) module implemented in the AMBER20 package. In addition, the trajectory in AMBER coordinate format (.mdcrd) file was converted into a PDB file using cpptraj in the AMBER20 package. Structural visualization and interaction analysis of IL-18 was performed using the Visual Molecular Dynamics (VMD) package (Humphrey, Dalke & Schulten, 1996).

### Statistical analysis

All the experiments were performed in triplicate. Statistical analyses were performed using SPSS version 26 (IBM, Armonk, NY, USA), and one-way analysis of variance (ANOVA) followed by Bonferroni or Least Significant Difference (LSD) multiple-comparison tests were applied to compare multiple groups. All comparisons were made with respect to the untreated groups. The *P*-value < 0.05 was considered statistically significant.

## Results

### Structural analysis of surface cysteines of IL-18

**Figure 1:**
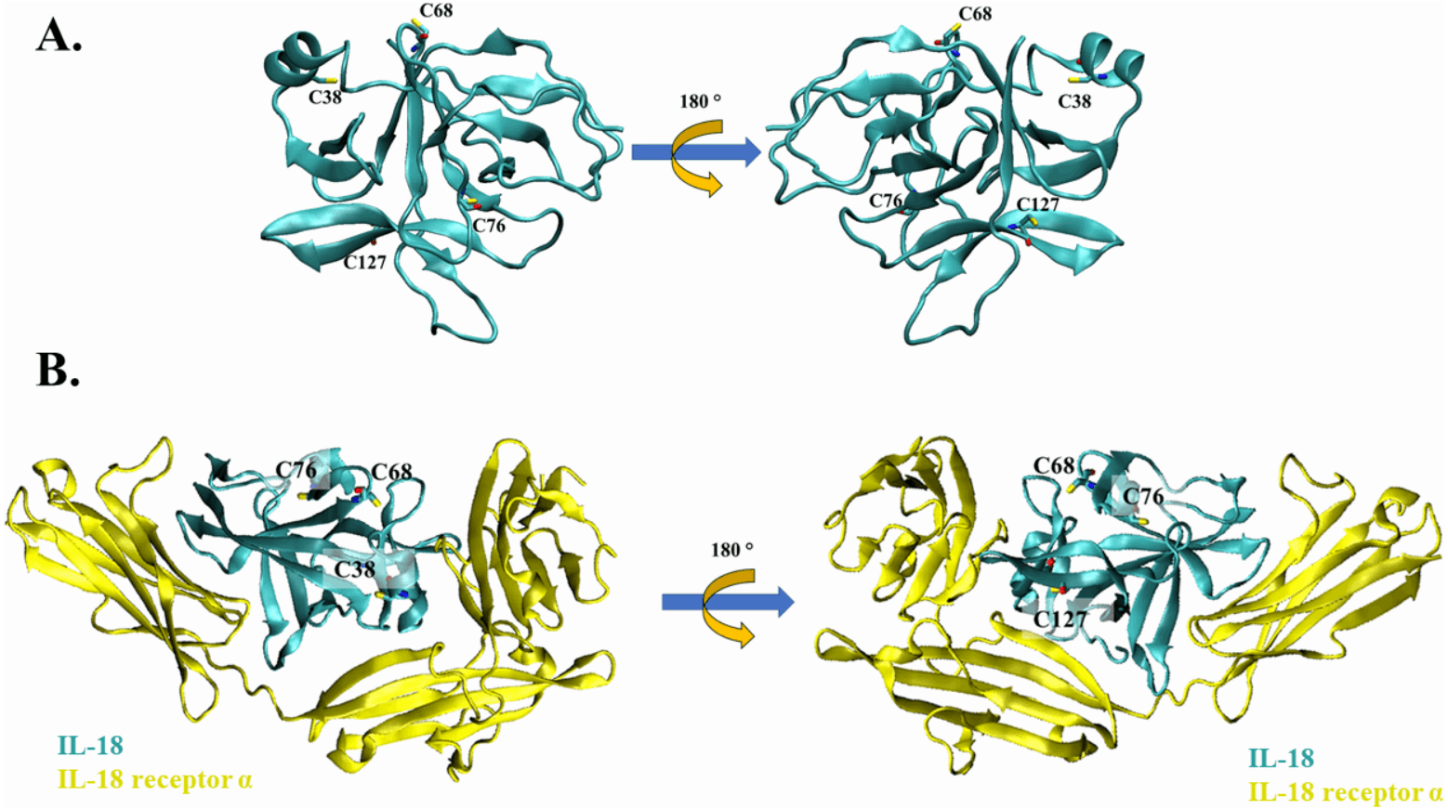
The crystal structure of wild-type IL-18 in complex with receptor. (A) The ribbon structure of mature human IL-18 (3WO3) is retrieved from Protein Data Bank. Surface cysteines mutated in this study are indicated. (B) The ribbon structure of IL-18 (cyan) with IL-18 receptor α (yellow).

Cysteine residues present on the surface of IL-18 were analyzed in a previous study (Yamamoto et al., 2004); however, the source was NMR-derived PDB structure (PDB ID: 1J0S) (Kato et al., 2003). Currently, human IL-18 is crystallized together with its receptors (Tsutsumi et al., 2014), allowing researchers to study the role of each amino acid residue of IL-18 in its interaction with the receptor. In this study, we used the crystal structure of human IL-18 in complex with IL-18 receptor α (PDB ID: 3WO3) to re-analyze the role of surface cysteines in their interaction with the receptor. As shown in Figs. 1A and 1B, the surface cysteines, including C38, C68, C76, and C127, were independent and did not interact with each other. The thiol side chain of most cysteines was directed toward the surface of the IL-18 molecule. No intramolecular interactions were observed, suggesting the role of these surface cysteines in intermolecular interactions. Additionally, none of the cysteine residues were observed to be important for binding with IL-18 receptor α (Fig. 1B). This is in accordance with the previous report on NMR-derived IL-18 structure, which showed that amino acid replacement did not disrupt the interaction between IL-18 and its receptors (Yamamoto et al., 2004). Therefore, we mutated the surface cysteines to improve our previously modified human IL-18 double mutant.

### Expression and refolding of recombinant IL-18 proteins

**Figure 2:**
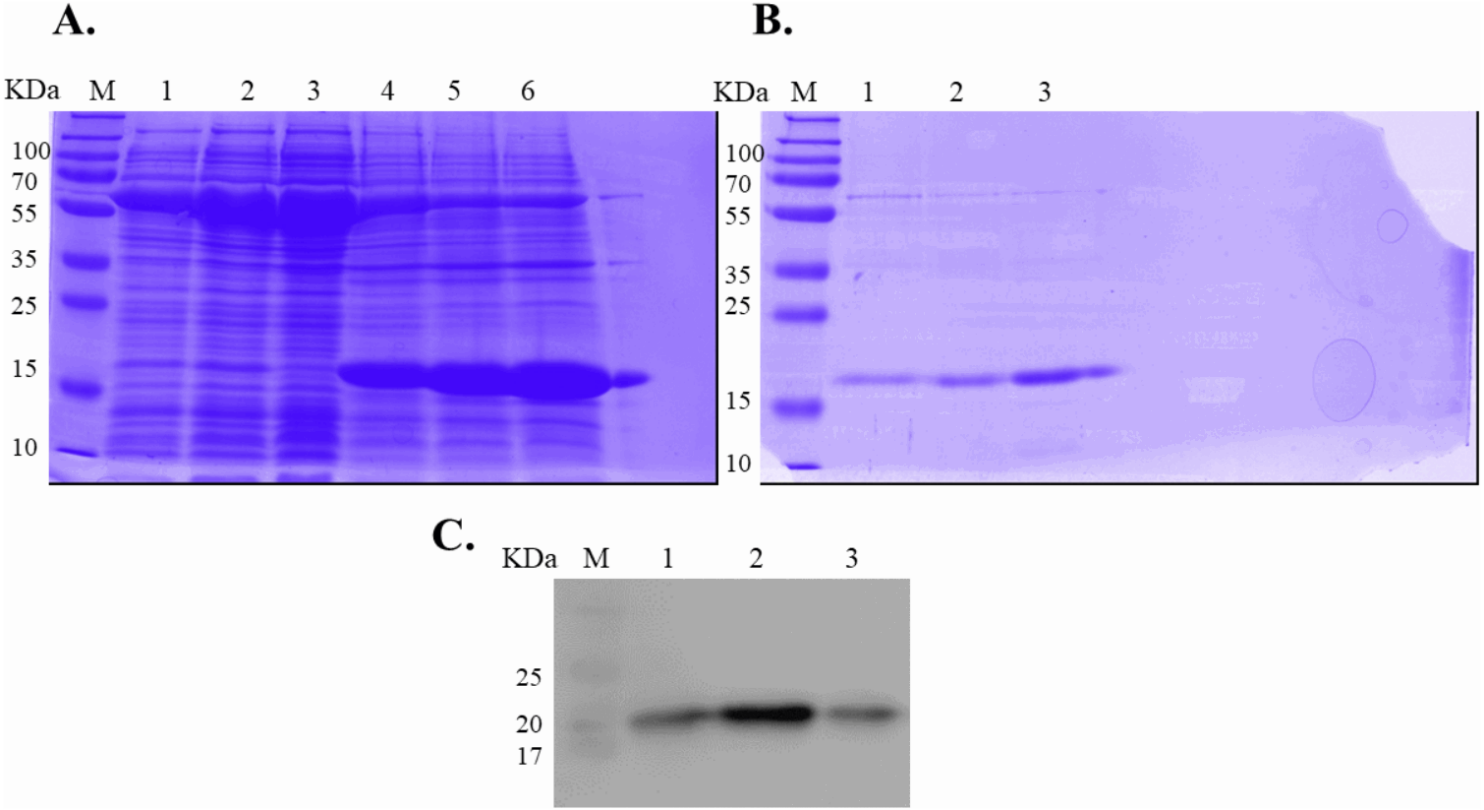
SDS-PAGE analysis of recombinant IL-18 before and after refolding. (A) SDS-PAGE showing soluble and insoluble fractions obtained after IL-18 expression in *E. coli*. Lane: M = protein marker, 1 = soluble fraction of wild-type (WT) IL-18, 2 = soluble fraction of mutant IL-18 DM, 3 = soluble fraction of mutant IL-18 DM1234, 4 = insoluble fraction of WT IL-18, 5 = insoluble fraction of mutant IL-18 DM, 6 = insoluble fraction of mutant IL-18 DM1234. (B) SDS-PAGE showing insoluble protein fractions after refolding. M = protein marker, 1 = IL-18 WT, 2 = IL-18 DM, 3 = IL-18 DM1234. (C) Western blot analysis of refolded IL-18 protein using anti-human IL-18 antibody. All samples were loaded onto 12% SDS-PAGE gel.

**Figure 3:**
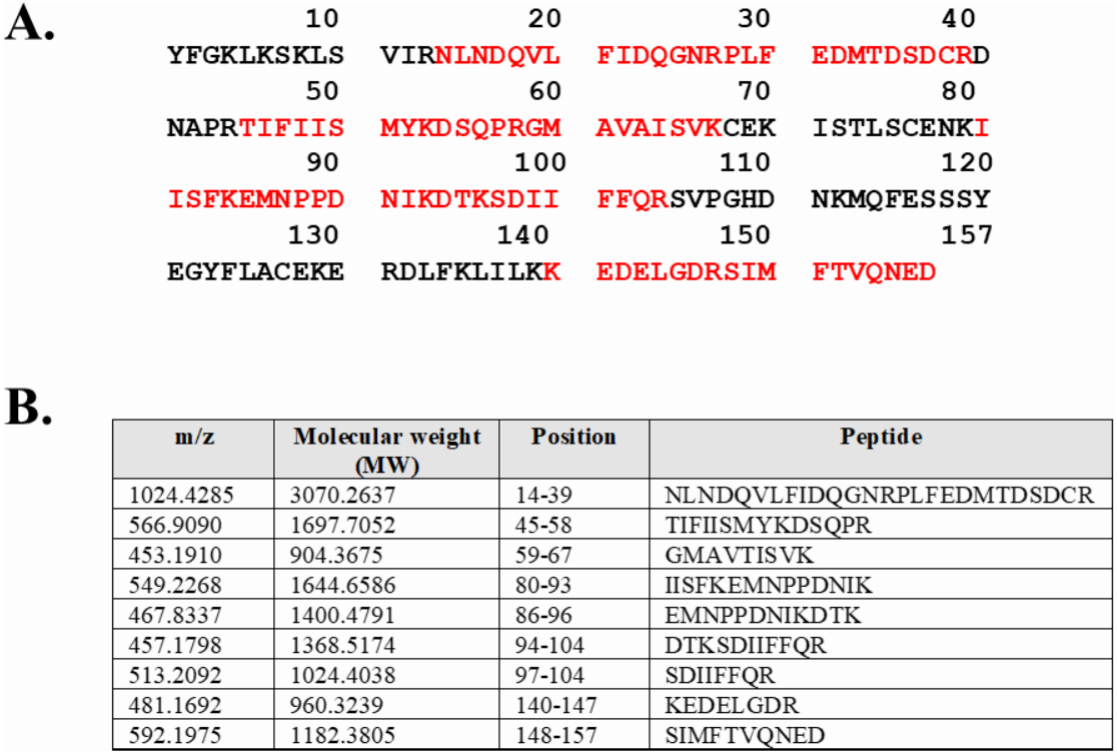
LC-MS/MS analysis of the refolded mature IL-18. (A) Tryptic peptide map of recombinant mature IL-18 expressed in *E. coli*. The identified peptides are shown in red. (B) Tryptic peptide fragments derived from IL-18.

Expression of WT and mutated IL-18 proteins was conducted using *E. coli* Rosetta2 (DE3) pLysS expression system. After cell lysis, the soluble fractions and the insoluble inclusion bodies of crude proteins were analyzed by SDS-PAGE. Figure 2 demonstrates that a protein band of approximately 20 kDa corresponding to the mature form of human IL-18 was detected in both the soluble fractions and inclusion bodies; the same protein band was not observed in control transformants with an empty vector (Fig. S1). However, more than 80% of the proteins were observed in inclusion bodies (Fig. 2A, lanes 4, 5, and 6), suggesting that human IL-18 was expressed primarily in insoluble form as inclusion bodies in *E. coli*. Therefore, stepwise refolding was performed to refold the IL-18 protein to its correct structure. SDS-PAGE analysis showed that the protein purity was greater than 95% after the refolding step; however, the yield was less than that of the starting source (Fig. 2B). Western blotting confirmed IL-18 specific protein bands using an anti-human IL-18 antibody (Fig. 2C). Further, we validated recombinant IL-18 by excising the protein band from the gel and subjecting it to LC-MS/MS analysis. Tryptic digestion of the protein provided 9 peptide fragments which covered about 48.0% of the pro-form of IL-18 or 58.8% of the mature IL-18 (Figs. 3A and 3B). These data suggest that the refolded IL-18 is the mature form of IL-18.

### IL-18 DM1234 showed lower aggregation capability and higher activity

**Figure 4:**
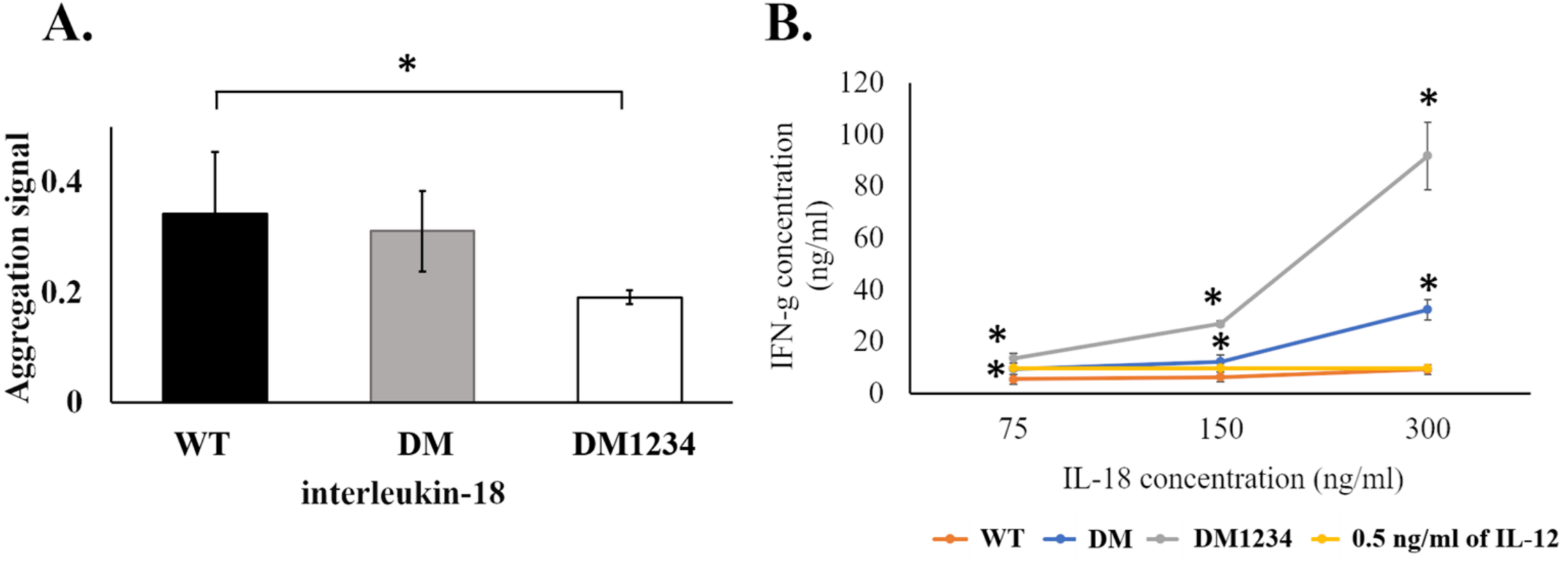
Protein aggregation and IFN-γ induction assays of wild-type and mutated IL-18. (A) Signal obtained from ProteoStat protein aggregation assay of each refolded IL-18. (B) IFN-*γ* induction assay with each refolded IL-18. NK-92MI cells were treated with various concentrations of IL-18 for 16–18 h. IFN-*γ* levels were measured in the supernatant by ELISA. All illustrated results represent the mean ± SD of three independent experiments. An asterisk (*) indicated *p* < 0.05.

To evaluate the effect of surface cysteine replacement with serine (C-to-S substitution) on IL-18 aggregation, oxidative stress was induced by shaking the refolded protein at 220 rpm in a shaker incubator. Heat (50 °C) was also used to increase the reaction energy. After 16–18 h of induction, the aggregated protein was measured using the ProteoStat protein aggregation assay. This method employs a fluorescent ProteoStat dye for protein aggregate detection, which is more sensitive than the oligomerization assay. Moreover, it is the only method that can detect protein aggregates at visible to sub-visible levels (Oshinbolu et al., 2018). The result demonstrated that protein aggregation was similar in IL-18 WT and IL-18 DM (Fig. 4A). However, IL-18 DM1234 showed a significant decrease in protein aggregation (Fig. 4A). C-to-S substitution lowered the aggregation ability of IL-18 DM1234 by approximately 1.79 and 1.63 folds compared to that of IL-18 WT and IL-18 DM, respectively (Data S1). Interestingly, the IFN-γ induction assay results demonstrated that the mutant IL-18 DM1234 showed a statistically increasing capability to stimulate IFN-γ production from NK-92MI cells (Fig. 4B). The IFN-γ levels induced by IL-18 DM1234 indicated higher efficacy of approximately 10.0 and 2.8 folds compared to that by IL-18 WT and IL-18 DM, respectively (Data S2).

### C-to-S substitution may improve the stabilization of IL-18 DM1234 for receptor binding

**Figure 5:**
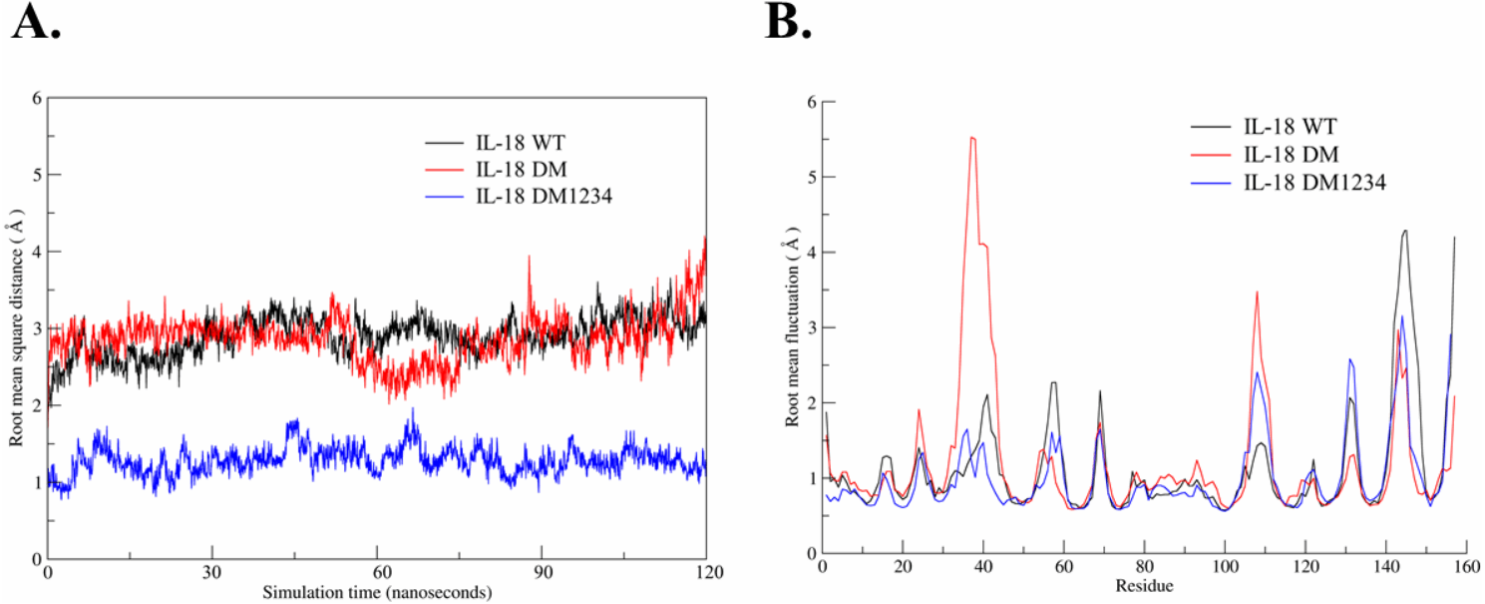
Root-mean-square deviation (RMSD) and root-mean-square fluctuation (RMSF) of wild-type and mutated IL-18. (A) RMSD values calculated for each IL-18 chain over the last 120 ns of the molecular dynamics (MD) simulations. (B) RMSF values of Cα carbon atoms of IL-18 amino acid residues during 120 ns of the MD simulations.

Based on the results of the IFN-γ induction assay, MD simulations were performed to investigate the role of C-to-S substitution on the structure of IL-18, which may affect the activity of this protein. Figure 5A shows the backbone root-mean-square deviation (RMSD) of each IL-18 protein with different patterns. The results demonstrated that 120 ns of MD simulation with physiological parameters was a sufficient time point for equilibration. Interestingly, IL-18 WT and IL-18 DM revealed similar RMSD values and patterns, whereas IL-18 DM1234 showed lower RMSD deviations (Fig. 5A). In addition to RMSD, MD simulation was used to track the flexibility of IL-18 as a root-mean-square fluctuation (RMSF) value. Figure 5B illustrates the degree of flexibility of amino acid sequences of the three IL-18 proteins. The results showed a similar RMSF value pattern for most amino acid sequences of the IL-18 proteins. However, there were two regions between 30–50 and 100–110 amino acid residues where IL-18 DM showed noticeably higher RMSF values compared with the other regions, indicating that these amino acids displayed greater flexibility.

**Figure 6:**
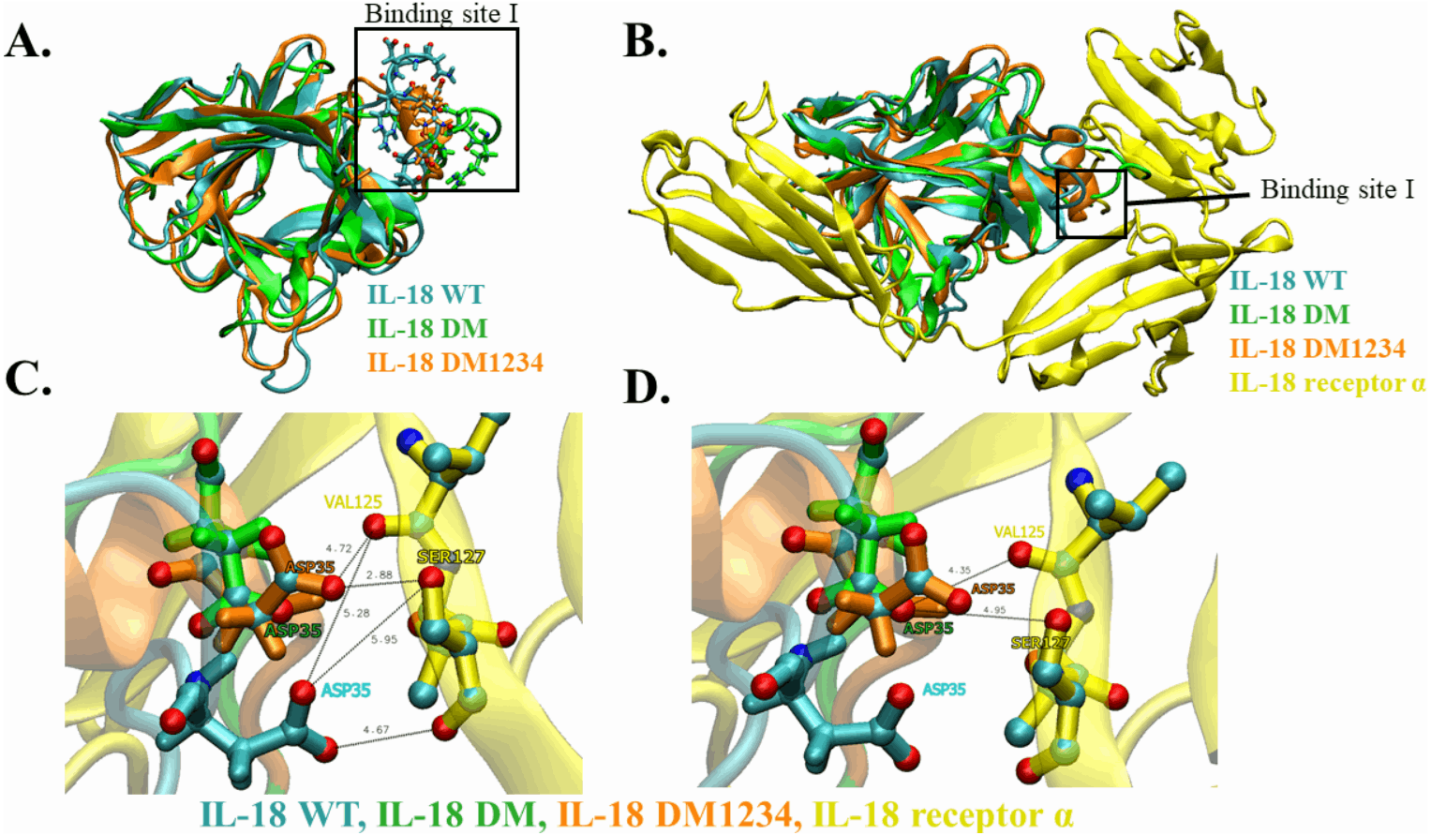
Structural analysis of IL-18 based on MD simulations. (A) Structural alignment of wild-type (WT) and mutated IL-18. The binding site I is indicated in the figure. (B) Altered regions in IL-18 DM1234 structure. The interface between binding site I of each type of IL-18 and its IL-18 receptor α is shown. (C) Interaction of D35 residue of IL-18 WT and IL-18 DM1234 with V125 and S127 residues of IL-18 receptor α. (D) Interaction of D35 of IL-18 DM with V125 and S127 residues of IL-18 receptor α. The distance between interacting amino acids is indicated in angstrom (Å).

WT and mutated IL-18 protein structures were visualized and aligned using the VMD program to determine differences in their conformation. The results showed an apparent conformational change in IL-18 DM1234 at the α-helix part of binding site I when compared to IL-18 DM (Figs. 6A and 6B). The binding site I comprise methionine 33 (M33) and aspartate 35 (D35), identified as essential residues for binding of IL-18 with IL-18 receptor α (Kato et al., 2003). Figure 6B demonstrates that the conformational alteration of IL-18 DM1234 resulted in a higher similarity of its binding site I region with IL-18 WT, which may improve the stabilization of IL-18 DM1234 for IL-18 receptors, similar to IL-18 WT structure. Additionally, looking deeper into the direction of amino acids in this area, we observed that D35 of IL-18 DM1234 changed its direction closer to valine 125 (V125) and serine 127 (S127) of IL-18 receptor α; this change was not observed in IL-18 WT (Fig. 6C). Structural alignment revealed that the distance between D35 of IL-18 DM1234 and V125 and S127 of the IL-18 receptor was in the range of 2.88–4.72 Å (Fig. 6B). On the other hand, D35 of IL-18 WT showed a higher distance of 5.28 and 5.95 Å  between V125 and S127, respectively (Fig. 6C). Moreover, D35 of IL-18 DM also showed increased length of these molecular pairs (4.35 and 4.95 Å  for V125 and S127, respectively) when compared to IL-18 DM1234 (Fig. 6D).

## Discussion

IL-18 has long been proposed as a potential candidate for cancer treatment. Many studies have revealed the role of this cytokine in inducing IFN-γ, which plays a vital role in anti-cancer immunity (Castro et al., 2018). However, the limitation of using IL-18 was demonstrated by its low impact in clinical trials (Robertson et al., 2006), which could be due to its aggregation-induced instability and increased levels of its natural inhibitor IL-18BP (Srivastava, Salim & Robertson, 2010). IL-18 has been modified mainly through site-directed mutagenesis, based on the existing knowledge of its binding to IL-18 receptors, to develop engineered variants showing enhanced activity and bioavailability (Kim et al., 2001; Kim et al., 2002; Kato et al., 2003; Yamamoto et al., 2004; Saetang et al., 2016).

In this study, we have attempted to improve our previously modified IL-18 DM protein, which is prone to aggregation at high concentrations (Fig. S2). Although the previous two mutations (E6K+T63A) could enhance the efficiency of IL-18, aggregation was still a problem because of the unpredictable activity and immunogenicity results. Loss of activity or induction of an unfavorable immune response was reported in several types of FDA-approved alpha-helical cytokines, such as IL-2 (Fatima et al., 2012), IFN-β-1b (Lipiäinen et al., 2015), and granulocyte colony-stimulating factor (G-CMS) (Krishnan et al., 2002) that were found to form multimeric precipitates under certain conditions, especially under physiological conditions. Yamamoto et al. (2004) reported that free surface cysteines of IL-18 (C38, C68, C76, and C127) might contribute to its aggregation based on computational structure predictions. Replacement of the four surface cysteines with serine and subsequent exposure to oxidative stress resulted in less aggregation than in the WT IL-18 (Yamamoto et al., 2004). Similar results were obtained in the present study after C-to-S substitutions in IL-18 DM, revealed by the low fluorescence signal of IL-18 DM1234 in protein aggregation assay compared to the WT and IL-18 DM. This suggests that surface cysteine of IL-18 contributes to intermolecular bonding, which is also observed in other cytokines (Karpusas et al., 1997; Lipiäinen et al., 2015). For example, a C-to-S substitution was introduced in IFN-β-1b to increase the stability of this cytokine, which is commercially used to treat relapsing forms of multiple sclerosis (Lin, 1998). Aggregation due to cysteine residues was also observed in IL-31, where the disulfide bond formation occurred at the intracellular level. Replacement of the cysteine residues with serine helped in improving the aggregation phenomenon (Shen et al., 2011). Interestingly, studies on human α-synuclein also emphasized the role of cysteine in protein stability. Aggregation of α-synuclein in Lewy bodies in midbrain dopamine neurons is usually associated with Parkinson’s disease. Replacement of tyrosine with cysteine in α-synuclein increased its aggregation rate under oxidative stress, leading to cellular toxicity, which may be associated with Parkinson’s disease (Zhou & Freed, 2004).

In addition to decreased aggregation rate under oxidative conditions, IL-18 DM1234 showed a higher ability to induce IFN- γ in NK-92MI cells than IL-18 DM. This may be caused by structural changes at the binding site I of IL-18 DM1234, resulting in a more suitable conformation for receptor binding. MD simulation revealed that altered positioning of residue D35 of IL-18 DM1234 near residues V125 and S127 of IL-18 receptor likely led to the formation of stronger hydrogen bonds between the molecules. Notably, this interaction has earlier been reported to play an essential role in stabilizing the α-helical structure of IL-18, which mediates IL-18 interaction with IL-18 receptor α (Tsutsumi et al., 2014). Another explanation for the improved IL-18 receptor binding may be increased surface polarity of IL-18 due to C-to-S substitutions because serine shows a lower hydrophilic index than cysteine (Kyte & Doolittle, 1982). The reduction in the aggregation rate could be another factor that enhanced the efficacy of this cytokine, possibly by increasing the active form of IL-18 DM1234 in the system. Therefore, C-to-S substitution enhanced the ability of IL-18 to activate NK-92MI either way by reducing protein aggregation or inducing IFN-γ secretion.

## Conclusion

In summary, we successfully produced IL-18 mutants using a heterologous *E. coli* expression system. Although the protein was expressed mainly as inclusion bodies, we yielded the soluble and active form of IL-18 by refolding in presence of urea. IL-18 DM1234 with C-to-S substitutions was demonstrated to be less prone to aggregation than the WT and IL-18 DM. The same mutations promoted the activation of IL-18 in the NK-92MI cells. A detailed mechanism of this scenario has not been elucidated; however, apparent structural changes in binding site I possibly led to a more appropriate conformation of IL-18 for receptor binding. Moreover, the enhancement in IL-18DM1234 activity may be associated with an increase in the active form of IL-18 due to decreased aggregation. Further studies to confirm this mechanism will be helpful in the development of engineered IL-18 proteins as prospective drugs for therapeutic use.

## Supplemental Information

10.7717/peerj.13626/supp-1 Supplemental Information 1 Aggregation assay

10.7717/peerj.13626/supp-2 Supplemental Information 2 Supplementary figures

10.7717/peerj.13626/supp-3 Supplemental Information 3 IFN-gamma induction assay

10.7717/peerj.13626/supp-4 Supplemental Information 4 Western blot with anti-IL-18 antibody

## Additional Information and Declarations

### Data Availability

The following information was supplied regarding data availability: Raw data are available in the Supplementary Files.
