# Supplementary material for: Surface cysteine to serine substitutions in IL-18 reduce aggregation and enhance activity
Source: PeerJ. 2022 Jul 5;10:e13626. doi: 10.7717/peerj.13626 (PMC9266699; doi:10.7717/peerj.13626)
Supplement: Supplemental Information 2 [file peerj-10-13626-s002.docx]

**Supplementary figures**


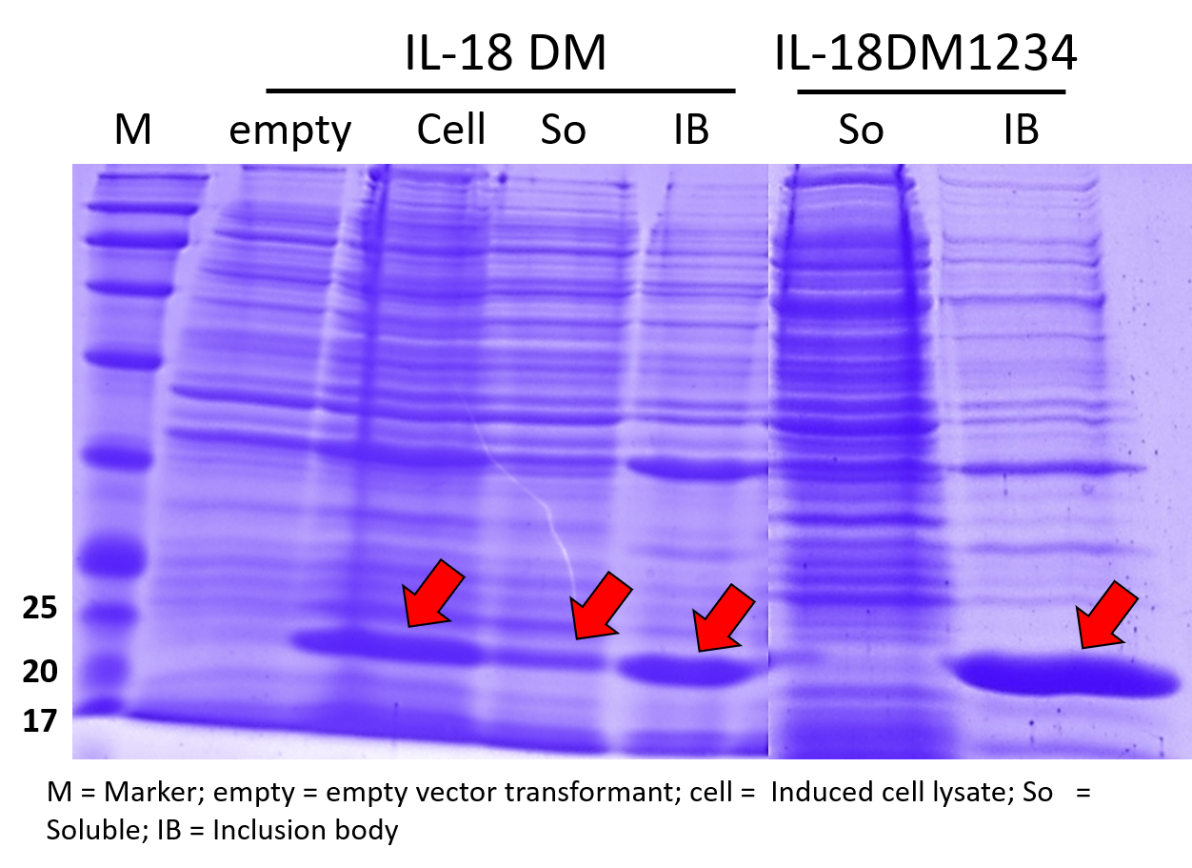


**Figure S1.** The expression of IL-18 DM and DM1234 using *E. coli* expression system.

**

**

**Aggregate form**

**Figure S2.** Western blot analysis of purified recombinant IL-18 from the previous work. An anti-human IL-18 antibody was used in this study. The arrow indicated the aggregated form of IL-18 at high concentrations.
