# Supplementary figures and images for: Surface cysteine to serine substitutions in IL-18 reduce aggregation and enhance activity
Source: PeerJ. 2022 Jul 5;10:e13626. doi: 10.7717/peerj.13626 (PMC9266699; doi:10.7717/peerj.13626)

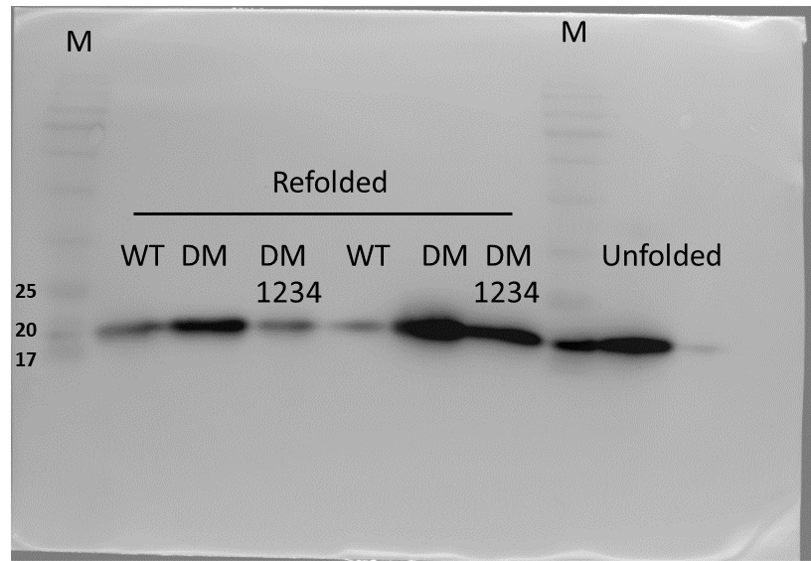

Supplement: Supplemental Information 4 [file peerj-10-13626-s004.png]
